# Comparison of endurance and electromyographic activity of core muscles in overhead athletes with and without scapular dyskinesis

**DOI:** 10.1038/s41598-026-56082-8

**Published:** 2026-05-30

**Authors:** Hadis Sabzikar Tahani, Hooman Minoonejad, Shahrzad Zandi, Ebrahim Ebrahimi

**Affiliations:** 1 Department of Sports Injury and Biomechanics, Faculty of Sport Sciences and health, University of Tehran, Tehran, Iran; 2 Department of Sport Injuries and Biomechanics, Faculty of Sport Sciences and Health, University of Tehran, Tehran, Iran

**Keywords:** Scapular dyskinesis, Core endurance, Electromyography, Overhead athletes, Anatomy, Health care, Medical research, Physiology

## Abstract

**Background:**

Scapular dyskinesis (SD) is a prevalent dysfunction among overhead athletes. Although the kinetic chain emphasizes the functional linkage of the core and shoulder musculature, limited evidence exists regarding the endurance and electromyographic (EMG) characteristics of core muscles in athletes with SD. This study aimed to compare core muscle endurance and EMG activity during closed kinetic chain tasks between overhead athletes with and without SD.

**Methods:**

Thirty female overhead athletes were divided into two groups: SD (n = 15) and without SD (n = 15), diagnosed using the Kibler lateral scapular slide test. Core endurance was assessed using the McGill Core Endurance Test, including the trunk flexor test, the trunk extensor test, and the right and left lateral plank. Surface EMG (ME6000, Finland) was used to record muscle activity of the erector spinae, multifidus, gluteus medius, transversus abdominis/internal oblique, external oblique, rectus abdominis, and serratus anterior during plank and push-up tasks. Independent t-tests and Cohen’s *d* were used to compare groups (*α* ≤ 0.05).

**Results:**

Athletes with SD demonstrated significantly lower endurance across all McGill tests (*p* ≤ 0.05, *d* = 0.81–0.88). EMG data showed reduced activation of the erector spinae, multifidus, gluteus medius, rectus abdominis, transversus abdominis/internal oblique, and serratus anterior muscles in the SD group during both tasks (*p* ≤ 0.05). No significant differences were observed in the external oblique activity.

**Conclusion:**

These findings highlight the importance of integrating targeted core stabilization and scapular control exercises into training and rehabilitation programs to optimize performance and prevent shoulder injuries in overhead athletes.

## Introduction

Shoulder pathologies occur mainly in overhead sports, where repetitive and high-velocity arm movements place significant stress on the shoulder complex ^1^. Scapular dysfunction, commonly referred to as scapular dyskinesis (SD), is one of the most prevalent and significant contributors to shoulder injuries, especially in overhead sports ^2^. SD refers to abnormal changes in the movement or position of the scapula, characterized by the protrusion of the inferior angle and medial border of the scapula away from the rib cage ^3^. Scapular motion and its dynamic stabilization are primarily governed by a group of muscles, including the trapezius, serratus anterior, pectoralis minor, levator scapulae, rhomboids, and teres major ^4^. Research has shown that individuals with SD often exhibit heightened activity in the upper trapezius, pectoralis minor, levator scapulae, and teres major, along with reduced activation of the middle and lower trapezius, serratus anterior, and rhomboid muscles ^5–7^. It has been reported that SD most frequently occurs in repetitive overhead sports (61%), especially baseball, volleyball, and handball ^8,9^. Some studies showed that overhead athletes with scapular dyskinesis have altered electromyographic (EMG) activity ^10^, exhibiting altered scapular kinematics in symptomatic dyskinesis ^11^. In addition, the overhead throwing motion is a fundamental movement pattern that involves transferring momentum from the pelvis and trunk to the throwing arm ^12^. This kinetic chain, which represents the interconnected segments from the lower limbs through the lumbopelvic complex to the upper extremity and relies on coordinated stability, strength, and endurance of core muscles, is essential for achieving both speed and accuracy in overhead throwing ^13,14^.

The core muscles encompass a diverse range of muscle groups that stabilize the shoulder girdle, pelvis, and spine, providing a pivotal foundation for limb movements ^15^. These muscles assist in controlling motion, transferring force and body weight, and distributing external loads throughout the body ^16^. Core muscle endurance plays a fundamental role in maintaining lumbopelvic stability during prolonged or repetitive athletic activities ^17^. In overhead athletes, insufficient core endurance may compromise proximal stability, leading to altered sequencing of movement and increased compensatory loading on the upper extremity ^18^. The global muscles of this region include the rectus abdominis, erector spinae, external obliques, and gluteal muscles, while the local muscles consist of the transversus abdominis and multifidus ^19^. It appears that the core muscles play a crucial role in stabilizing the lumbopelvic region, and evidence suggests that the stability of this area influences the activation of muscles in other parts of the body ^20,21^. Kibler believes that there is an association between core muscles and the scapula based on the concept of muscular synergy ^22^. According to this, Kibler suggests that various muscles in the body work together in a chain-like manner to produce coordinated and balanced movements ^23^. Within the scapular kinetic chain, the core and trapezius muscles play a fundamental role. The core muscles provide stability and support to the shoulder girdle complex, contributing to the functional stability of the scapulothoracic articulation and associated ligamentous structures ^24^. Although overhead sports involve explosive and high-velocity trunk actions, trunk muscular endurance plays a fundamental role in maintaining lumbopelvic stability and ensuring efficient force transmission throughout repeated overhead movements ^25,26^. Insufficient endurance may compromise proximal stability within the kinetic chain, potentially contributing to altered scapular mechanics and scapular dyskinesis. Additionally, sex-related differences in neuromuscular function have been reported in both core endurance and EMG activity. Female athletes often demonstrate lower absolute trunk strength but may rely more on endurance-based stabilization strategies compared to males ^27,28^.

In addition, previous studies have examined the kinetic chain in overhead athletes and the relationship between scapular muscles and core musculature ^20,29^, there is a notable lack of research specifically addressing core muscle endurance and EMG activity in relation to SD. Therefore, there is a critical need to investigate EMG activity and endurance of core muscles in overhead athletes with and without SD to elucidate their role in athletic performance, inform targeted training interventions, and support injury prevention and rehabilitation strategies. It was hypothesized that overhead athletes with SD would demonstrate reduced core muscle endurance and altered EMG activity of core and scapular stabilizing muscles during closed kinetic chain tasks compared to athletes without SD.

## Material and methods

This cross-sectional study included female students from overhead sports including handball, volleyball, and badminton with and without SD from the Faculty of Physical Education and Sport Sciences at the University of Tehran. These sports were selected due to their shared reliance on repetitive overhead movements and high kinetic chain demand. Based on a study ^30^, 30 participants were chosen using G. Power software, ensuring a 0.88 test power, an effect size of 0.84, and a significance level of 0.05. They were divided into SD (N = 15) and without SD (WSD) groups (N = 15). Participants were included if they were female students aged 18 to 35 years, engaged in overhead sports for at least six months, and had no history of shoulder, abdominal, or lumbar trauma or surgery. In addition, participants with scapular dyskinesis were required to demonstrate the condition in both static and dynamic positions according to the Kibler test ^31^. In contrast, participants in the without SD group were required to show bilateral normal scapular motion. All participants were required to provide written informed consent to take part in the study. Participants were excluded if they had severe spinal deformities such as kyphosis or scoliosis (New York test score ≥ 5) ^32^, had performed heavy physical activity within 48 h prior to testing, experienced pain in the shoulder or lumbar region on the day of testing, or withdrew consent to participate in the study. At first, for ethical considerations based on the Declaration of Helsinki, all study participants were informed of all stages of the study, and then written informed consent was obtained. Secondly, individuals were informed that in the event of any issues during the tests, the examiner, a sports science student, would take all necessary actions. All steps were explained to the participants. It is worth noting that all tests were conducted at Tehran University’s laboratory. Before starting the investigation, study approval was obtained from the Biomedical Research Ethics Committee of the University of Tehran (Ethics code: IR.UT.SPORT.REC.1402.062).

### Procedures and data reduction

The Kibler lateral scapular slide test (1998) was the initial method for evaluating scapular dyskinesis. The test involved marking the inferior angle of the scapula on the skin using a marker and measuring its distance from the adjacent vertebra in three standing positions: arms at sides, hands on hips with thumbs posterior and fingers anterior, and arms abducted 90 degrees with thumbs downward. The average of three repeated measurements on each side was calculated. A difference greater than 1.5 cm between the two sides signifies a positive test result ^31^. Fatigue-based or repetitive elevation tasks were not included to ensure standardization of measurement and to minimize variability between participants. Although SD patterns were visually observed, participants were not stratified by subtype due to sample size limitations, and SD was analyzed as a single clinical condition.

To assess core muscle endurance, the McGill Core Endurance Test was used ^33^. The protocol included four tests designed to comprehensively assess torso strength through isometric muscle endurance. The Trunk Flexor Test (TFT) was employed to evaluate the endurance of the anterior core musculature, primarily the rectus abdominis ^34^. In the TFT, participants assumed a sit-up position with the back resting against a jig inclined at 60° from the floor, hips and knees flexed at 90°, arms crossed over the chest, and feet secured. The jig was then pulled back 10 cm, and participants were instructed to maintain the isometric posture for as long as possible. The test was stopped when any portion of the participant’s back contacted the jig, indicating failure ^35^. The total duration that the position was maintained was recorded in seconds, providing a quantifiable measure of trunk flexor endurance. The Trunk Extensor Test (TET) assesses the endurance of the erector spinae and multifidus muscles. During the test, the clinician stands to the side of the participant’s torso to ensure proper alignment. The participant’s upper body is positioned cantilevered over the end of the test bench, with the pelvis, knees, and hips securely stabilized. The upper limbs are crossed over the chest, with the hands resting on the opposite shoulders. The test was stopped when the participant is unable to maintain the upper body in a horizontal position, indicating failure ^35^. The Lateral Musculature Tests (LMT) are used to evaluate the obliques, transverse abdominals, and quadratus lumborum muscles. The test is performed separately for the left and right sides. The participant lies in a full side-bridge position, with the legs extended and the top foot placed in front of the lower foot for additional support. The participant supports themselves on one elbow and their feet while lifting the hips off the floor to maintain a straight line from head to toe. The uninvolved arm is held across the chest, with the hand placed on the opposite shoulder. A test failure occurs when the participant loses the straight-back posture or when their hips return to the ground. Inability to assume the correct position indicates significant weakness in the lateral core musculature ^35^. EMG activity of the erector spinae, multifidus, gluteus medius, transversus abdominis/internal oblique, external oblique, rectus abdominis, and serratus anterior was recorded. The gluteus medius and serratus anterior were included due to their critical roles in kinetic chain function. The gluteus medius contributes to pelvic stability and force transmission from the lower extremity to the trunk ^36^, while the serratus anterior is essential for scapular upward rotation and stabilization during upper limb loading tasks ^37^. Although the trapezius muscle plays a fundamental role in scapular control, it was not included in the present study. Additionally, inclusion of all trapezius subdivisions would have increased protocol complexity and participant burden during bilateral EMG assessment.

**Fig. 1:**
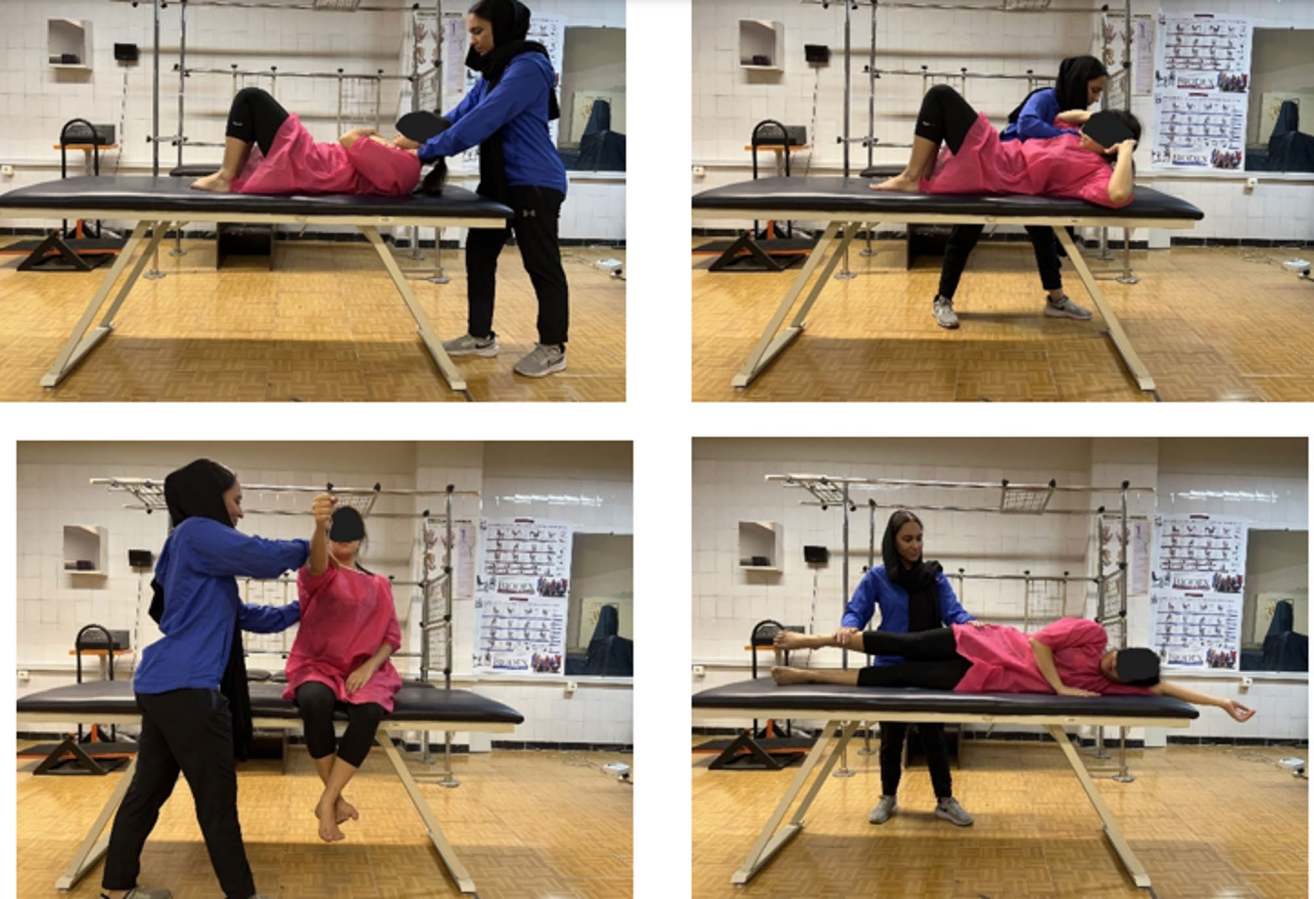
The maximum voluntary isometric contraction of core muscles.

To assess the electrical activity of the core muscles, a ME6000 surface electromyography (EMG) system (Megawin, Finland) was used. This 16-channel device is capable of wireless connection to a computer via electromagnetic waves, allowing for data collection. Consequently, by securing the device in its special belt around the participant and placing the EMG system inside the belt’s compartment, it can also be used during field-based and dynamic positions. For each muscle, two electrodes (positive and negative) were placed on the skin 2 cm apart, along with a reference electrode, following the SENIAM (Surface Electromyography for the Noninvasive Assessment of Muscles) ^38,39^. Electrode placement was as follows: erector spinae, two fingerbreadths lateral to L1; lumbar multifidus, approximately 2–3 cm lateral to the spinous process at the L5 level, in the region of the L5-S1 facet joint; gluteus medius, midpoint between the iliac crest and greater trochanter; transversus abdominis/internal oblique, 2 cm medial and inferior to the anterior superior iliac spine; external oblique, along a line from the lowest point of the costal margin to the contralateral pubic tubercle; serratus anterior, approximately over the mid-axillary line on the lateral thoracic wall, over the 5th to 7th ribs, at the level of the scapular inferior angle; and rectus abdominis, 1 cm below the umbilicus and 2 cm lateral to the midline ^40^. To assess maximum voluntary isometric contraction (MVIC), participants performed three 20-s trials, divided into two 7-s intervals, against manual resistance applied by the examiner. For data processing, the first and last 2 s of each trial were excluded, and the middle 3 s were analyzed. Although 20-s isometric contractions exceed typical maximal strength testing durations, this protocol was implemented to ensure stabilization of the EMG signal and to allow extraction of a consistent middle segment for analysis. Only the central portion of each contraction was used for processing to minimize the influence of initial and terminal variability. Specific MVIC maneuvers were as follows: lumbar multifidus and erector spinae, trunk extension in the prone position; gluteus medius, side-lying hip abduction against manual resistance at the ankle; rectus abdominis, attempted sit-up against manual resistance at the shoulders; external oblique, attempted rotational sit-up bringing the right shoulder toward the left knee (and vice versa) while the examiner stabilized the contralateral shoulder; and transversus abdominis, abdominal hollowing maneuver (Fig. 1) ^40^. This protocol ensured reliable, standardized measurement of core muscle activation during isometric contractions.

**Fig. 2:**
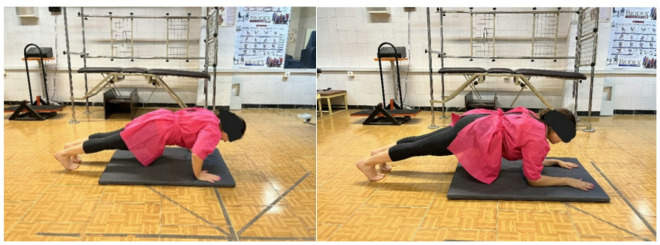
Push-up and plank tasks.

After a 10-min rest period, participants were prepared to perform Push-up and plank tasks. Push-up Task: Participants began in a position with elbows fully extended, thumbs aligned directly under the shoulders, and hands placed at shoulder width (Fig. 2). The body was lowered in a straight line from feet to shoulders until the elbows reached 90°, and then pushed back to full elbow extension following a 2–1-2 tempo ^41^. Participants repeated this task for 20 s. Any participant reporting discomfort or pain during the movement was withdrawn from the study.

Plank Task: Participants assumed a plank position with elbows flexed at 90°, ensuring their forearms and toes were in contact with the ground, while maintaining a straight body alignment (Fig. 2). Participants were instructed to maintain a neutral spine and stable plank position, avoiding pelvic drop or trunk rotation. Standardized verbal instructions were provided by the same examiner, and no muscle-specific coaching was given during testing. A familiarization trial was performed prior to data collection to ensure correct execution of the task and reduce learning effects. Each participant held this position for 20 s, with a 120-s rest, completing the task twice ^42^. During this stage, electrode placement was also checked and secured to ensure accurate sEMG recordings. It should be noted that all data were first standardized, and a band-pass filter with a cutoff frequency of 20 Hz and 450 Hz, using a 4th-order Butterworth filter, was applied. The maximum voluntary contraction (MVC) of each muscle was then calculated, and all EMG signals were normalized to the respective maximum MVC values. Subsequently, the root mean square (RMS) was computed using a 100-ms window, and the EMG signals were segmented into 2-s epochs. Due to the specific tasks selected for this study, the mean value over the 2-s segments was ultimately used for analysis.

### Data analysis

For data analysis, descriptive statistics methods were used to calculate the mean and standard deviation of height, weight, and age. The Shapiro–Wilk test was used to assess the normality of the data. Independent T-tests were used for parametric data with an alpha level of ≤ 0.05. Additionally, Cohen’s d was used to evaluate the effect size of the variables, with values below 0.5 considered small, between 0.5 and 0.8 considered medium, and values above 0.8 regarded as large.

## Results

The Shapiro–Wilk test results indicated that the data followed a normal distribution. Table 1 displays the demographic characteristics of participants in both groups. No significant differences were found between the SD and WSD groups in age, height, weight, or body mass index (all p > 0.05), indicating that the groups were comparable at baseline.

**Table 1:** Demographic characteristics of overhead athletes with SD and WSD.

Data	Group	Mean ± SD	*P*-value
Age (year)	SD	22.26 ± 3.67	0.395
	WSD	23.6 ± 4.71	
Height (cm)	SD	163.53 ± 5.98	0.330
	WSD	165.4 ± 7.03	
(kg)	SD	56.00 ± 6.53	0.208
	WSD	59.73 ± 5.28	
BMI (kg/m^2^)	SD	20.87 ± 1.36	0.968
	WSD	21.9 ± 2.4	

SD; scapular dyskinesis, WSD; without scapular dyskinesis, BMI; body mass index.

The results of Table 2 showed a significant difference in core endurance between athletes with and without SD (P ≤ 0.05), with the WSD group exhibiting superior endurance across all assessed tests. Also, the effect size analysis (Cohen’s d) revealed values of 0.885, 0.818, and 0.809, indicating a large effect of SD on core endurance in the side plank tests (both sides) and the trunk extensor test, respectively.

**Table 2:** Comparison of core muscle endurance between overhead athletes with SD and WSD assessed using the McGill Core Endurance Test.

Variable	Group	Mean ± SD (Seconds)	t value	Cohen’s d	*P*-value
TFT	SD	78.8 ± 39.06	2.104	0.768	0.046^*^
	WSD	116.66 ± 57.74			
TET	SD	115.93 ± 58.00	2.217	0.809	0.036^*^
	WSD	171.6 ± 78.06			
LMT (Left)	SD	43.66 ± 11.84	2.240	0.818	0.037^*^
	WSD	61.60 ± 28.65			
LMT (Right)	SD	40.93 ± 10.72	2.423	0.885	0.024^*^
	WSD	54.66 ± 19.15			

TFT; trunk flexor test, TET; trunk extensor test, LMT;l lateral musculature tests,

* Significant difference.

The results of Table 3 demonstrated a significant difference in core muscle EMG activity between individuals with and without SD during the plank task (P ≤ 0.05). However, no significant difference in the EMG activity of the external oblique muscle was shown (P ≥ 0.05). Furthermore, according to Cohen’s d results, the effect size analysis yielded values of 0.809, 0.845, 0.965, and 0.976, indicating a large effect of SD on the activity of the erector spinae, multifidus, gluteus medius, and serratus anterior muscles during the plank task.

**Table 3:** Comparison of EMG activity of core and scapular stabilizing muscles during the plank task in athletes with SD and WSD.

Variable	Group	Mean ± SD (MVIC%)	t value	Cohen’s d	*P*-value
Erector spinae	SD	2.79 ± 1.57	2.215	0.809	0.040^*^
	WSD	5.2 ± 3.9			
Multifidus	SD	4.01 ± 1.51	2.315	0.845	0.033^*^
	WSD	6.53 ± 3.93			
Gluteus medius	SD	4.57 ± 2.59	2.643	0.965	0.013^*^
	WSD	8.1 ± 4.47			
External obliques	SD	22.71 ± 9.3	0.509	0.186	0.614
	WSD	24.55 ± 10.46			
Transversus abdominis/internal oblique	SD	25.27 ± 7.69	2.106	0.769	0.044^*^
	WSD	32.26 ± 10.3			
Rectus abdominis	SD	24.81 ± 9.52	2.117	0.773	0.043^*^
	WSD	32.27 ± 9.77			
Serratus anterior	SD	26.22 ± 12.11	2.673	0.976	0.012^*^
	WSD	37.49 ± 10.93			

* Significant difference.

The results of Table 4 showed a significant difference in core muscle EMG activity between individuals with and without SD during the push-up task (P ≤ 0.05). However, no significant difference in the EMG activity of the external oblique muscle was shown (P ≥ 0.05). Additionally, according to Cohen’s d results, the effect size analysis revealed values of 0.900 and 0.988, indicating a large effect of SD on the activity of the erector spinae and rectus abdominis muscles during the push-up task.

**Table 4:** Comparison of EMG activity of core and scapular stabilizing muscles during the push-up task in athletes with SD and WSD.

Variable	Group	Mean ± SD (MVIC%)	t value	Cohen’s d	*P*-value
Erector spinae	SD	3.32 ± 1.44	2.465	0.900	0.025^*^
	WSD	6.63 ± 4.99			
Multifidus	SD	3.35 ± 1.21	2.136	0.780	0.047^*^
	WSD	5.24 ± 3.2			
Gluteus medius	SD	3.6 ± 2.32	2.064	0.754	0.049^*^
	WSD	5.24 ± 2.01			
External obliques	SD	13.46 ± 4.02	0.183	0.067	0.857
	WSD	13.84 ± 7.11			
Transversus abdominis/internal oblique	SD	12.77 ± 7.33	2.116	0.773	0.043^*^
	WSD	18.75 ± 8.13			
Rectus abdominis	SD	11.95 ± 10.51	2.707	0.988	0.011^*^
	WSD	21.94 ± 9.67			
Serratus anterior	SD	19.12 ± 6.21	2.186	0.798	0.037^*^
	WSD	24.04 ± 6.12			

* Significant difference.

## Discussion

This study found significant differences in core endurance and core muscle activity during push-ups and planks between individuals with and without SD. Core endurance allows prolonged stabilization of the trunk, enabling efficient and continuous force transfer during athletic and daily activities ^43^. Some studies suggest an indirect but functional connection between the core muscles and the upper extremity ^44,45^, mediated through the kinetic chain and muscular sling systems ^46^. This relationship is largely attributed to the thoracolumbar fascia, which serves as a key structure for transmitting force and energy between the lower and upper limbs ^47^. By linking deep trunk muscles such as the multifidus with the internal oblique and transversus abdominis, this fascia provides three-dimensional spinal stability and contributes to both core and shoulder function ^48^. Hence, our results were consistent with some studies. For instance, Atta et al. (2018) found a significant positive correlation between core endurance and scapular muscle function, indicating that greater core endurance was associated with better scapular muscle performance in individuals with chronic shoulder pain ^49^, while Gunaydin (2021) reported a similar association in elite amputee football players ^50^. In contrast, Ozturk et al. (2023) did not observe differences in trunk extensor endurance between athletes with and without SD ^51^. Although both studies used isometric endurance assessments, differences in participant characteristics, sport-specific adaptations, and study populations may explain the discrepancy between findings. In addition, the inclusion of multiple core muscle assessments in the present study may have provided greater sensitivity in detecting functional differences associated with SD.

Additionally, the SD group exhibited lower EMG activity in the core muscles compared to the group without SD in push-up and plank tasks. Specifically, significant differences in muscle activation were observed in the erector spinae, multifidus, gluteus medius, rectus abdominis, transversus abdominis/internal oblique, and serratus anterior muscles. Core muscle weakness and reduced activation were observed in athletes with SD, indicating an association between trunk muscle function and scapular control ^2^. Impairment in core function can disrupt the kinetic chain of force transmission, leading to altered movement patterns, decreased athletic performance, and an increased risk of injury ^52,53^. Another possible explanation for the increased activity of the muscles in this region in the WSD group relates to the generation and transmission of force produced by the trunk and lower limbs, which contributes to proper movement mechanics and performance in overhead athletes. According to Kibler et al. (2013), the trunk and lower extremities generate more than 50% of the kinetic energy transferred to the upper extremities ^54^, particularly to the arms. The legs and trunk develop this force through ground reaction forces, creating a stable base that enables efficient mobility and coordinated upper-limb motion ^55^.

Regarding the plank task, it is a closed kinetic chain movement that engages the entire body. Because the plank involves supporting body weight through the upper limbs, it places load on the scapular muscles, thereby activating both the scapular and core muscles simultaneously ^56^. In a study by Cortell-Tormo et al. (2016), it was found that scapular positioning significantly influences core muscle activation, enhancing their engagement ^57^. Overall, several studies support that the plank is a traditional core exercise that not only increases activation of the core muscles but also recruits the scapular muscles ^56,58,59^, which our results were aligned with. Another closed kinetic chain movement of this study was the push-up. Marcolin et al. (2015) examined muscle activity during the push-up and found that the pectoralis major, triceps brachii, serratus anterior, and anterior deltoid were the most active muscles ^60^. In the present study, the serratus anterior was specifically assessed due to its functional overlap and interaction with the external oblique. This muscle is a key scapular stabilizer and plays a role in scapular dyskinesis, and previous research has shown its high activation during push-up movements ^61,62^.

During push-ups performed on different surfaces, the erector spinae and internal obliques act as global stabilizers, while the rectus abdominis functions as a global mobilizer, with their activation patterns reflecting these roles ^63^. Anderson et al. (2013) showed that during push-ups, the internal oblique acts as a global stabilizer resisting core movement, while the erector spinae functions as both a local and global stabilizer, maintaining spinal stability and posture to support force generation by the primary movers ^64^. The possible mechanisms underlying these results can be examined from the perspective of the muscles engaged during these tasks. For erector spinae, lower activity in the SD group may be attributed to the role of the trapezius–serratus anterior force couple in providing scapulothoracic joint stability, which is essential for overall trunk and core stabilization ^65^. In addition, a significant relationship has been observed between the activation of the gluteus medius, lower trapezius, and serratus anterior in young baseball players ^66^. Kibler reported that weakness of the gluteus medius reduces force transmission to the shoulder ^54^. Given that in the present study, this muscle was engaged during a closed kinetic chain task, it can be considered that the gluteus medius plays a significant role in the kinetic chain and force transfer, resulting in higher activation in healthy athletes without SD. In regard to the rectus abdominis, it has been reported a significant correlation between trunk flexor endurance, including the rectus abdominis, and scapular muscle endurance ^67^. Similarly, another study identified early activation of the rectus abdominis preceding upper limb movements in specific directions ^68,69^. This study highlights the importance of core endurance and core muscle activation in the prevention and management of SD, particularly in overhead athletes. Reduced activity and endurance of key core muscles may impair the kinetic chain, disrupting force transmission from the lower to upper extremities and increasing the risk of shoulder dysfunction and injury. Clinically, these findings suggest that targeted core programs should be incorporated into rehabilitation and training protocols for athletes with or at risk of SD to optimize performance and reduce injury risk.

### Limitations

This study has several limitations that should be considered when interpreting the findings. First, the relatively small sample size may have limited statistical power and the ability to detect subtle associations. In addition, the inclusion of athletes from different overhead and racket sports may have introduced heterogeneity due to sport-specific movement patterns and loading demands, potentially increasing variability in the outcomes. Although this approach may enhance the generalizability of the findings to a broader athletic population, a more homogeneous sample might have provided more precise estimates. Furthermore, the plank and push-up tasks are closed kinetic chain exercises and may not fully represent the open-chain, high-velocity movements commonly observed in overhead sports. Therefore, the ecological validity of the findings for sport-specific performance is limited. Another limitation of this study is that SD was assessed only under static and controlled arm positions using the Kibler lateral scapular slide test. Fatigue-induced or repetitive overhead elevation conditions, which may further reveal dynamic scapular control deficits, were not included. A methodological limitation relates to the MVIC protocol, where 20-s isometric contractions were used for EMG normalization. While this approach is common in EMG studies to ensure signal stabilization, longer rest intervals between maximal contractions are typically recommended in maximal strength testing protocols to fully avoid fatigue effects. Also, residual fatigue effects from the McGill Core Endurance Test cannot be completely ruled out and may have influenced subsequent EMG measurements. Although trunk explosiveness is highly relevant in overhead sports, this study focused on endurance capacity as a foundational neuromuscular component of kinetic chain stability. Another limitation of this study is that although EMG was recorded bilaterally, potential differences between dominant and non-dominant sides were not analyzed separately. In addition, another limitation of this study is the absence of trapezius muscle assessment (upper, middle, and lower), which plays a key role in scapular control. A limitation of this study is the absence of muscle onset timing analysis, which could provide additional insight into neuromuscular coordination and activation sequencing within the kinetic chain. Finally, the cross-sectional design precludes any inference of causality between core musculature and scapular positioning. Future research with larger, sport-specific cohorts and more objective biomechanical assessments is warranted to confirm and extend these findings.

## Conclusion

Athletes with SD exhibited decreased trunk muscle endurance and reduced activity in the erector spinae, multifidus, gluteus medius, rectus abdominis, transversus abdominis/internal oblique, and serratus anterior during closed kinetic chain tasks. Therefore, a relationship was demonstrated between SD and decreased trunk muscle endurance and muscle activity.

## Data Availability

The datasets generated during the current study are available from the corresponding author on reasonable request.
